# Supporting movement and physical activity in people with psychosis: A qualitative exploration of the carer perspective

**DOI:** 10.1177/00207640241277166

**Published:** 2024-09-04

**Authors:** Rowan Diamond, Felicity Waite, Anne-Marie Boylan, Alice Hicks, Thomas Kabir, David Shiers, Daniel Freeman

**Affiliations:** 1 Oxford Cognitive Approaches to Psychosis (O-CAP), Department of Experimental Psychology, Radcliffe Observatory Quarter, University of Oxford, Oxford, UK; 2 Oxford Health NHS Foundation Trust, UK; 3 Nuffield Department of Primary Care Health Sciences, University of Oxford, UK; 4 The McPin Foundation, London, UK; 5 Psychosis Research Unit, Greater Manchester Mental Health NHS Trust, UK; 6 University of Manchester, UK; 7 School of Medicine, Keele University, UK

**Keywords:** Sedentary, exercise, schizophrenia, family, caregiver

## Abstract

**Background:**

The need to increase exercise and decrease sedentary behaviour in people diagnosed with psychosis is well-recognised.

**Aims:**

We set out to explore caregivers’ perspectives on what supports and prevents physical activity, and how to use carers’ support most effectively.

**Method:**

Fourteen caregivers of people diagnosed with psychosis were interviewed. Data were analysed using reflexive thematic analysis, in collaboration with caregivers.

**Results:**

Four themes were developed, the first flagging the importance of physical activity, then the others calling for action: (a) Physical inactivity matters: carers are keen to support efforts to increase physical activity in their family or friends because of the enormous impact physical inactivity has on patients, and consequently on carers themselves, such as social isolation and reduction in their own activity. (b) Tell us: without being well-informed about how to help, carers can feel like they are powerless to stop a ‘slow suicide’ or ‘decline’ in patients. (c) Listen to us: through knowing their family and friends well, carers are able to identify important changes in patients and identify successful motivators for them, but these insights can feel uninvited. (d) Ask us: being invited to support activity as a partner in a patients’ care is desirable but having offers of help rejected can “demotivate the motivator.”

**Conclusions:**

Caregivers described strong motivation to help patients to be more physically active but can feel that their support is overlooked and under-used by services. Clinical recommendations for carer involvement in physical activity interventions are offered.

## Introduction

People diagnosed with psychosis tend to spend less time exercising and more time being sedentary than other people in the general population (Stubbs, Firth, Berry, et al., 2016; Stubbs, Williams, Gaughran, et al., 2016). This inactivity contributes to the over-representation of preventable health conditions such as cardiovascular disease and metabolic disorder (De Hert et al., 2009) and to the lower life expectancy for people with severe mental illness (Hjorthoj et al., 2017). Inactivity may also contribute to cognitive impairment and depressive symptoms in people with mental disorders (Firth et al., 2017; Rosenbaum et al., 2014). Whilst attempts have been made in the past to help people with psychosis to increase their physical activity, they have often not been engaging enough (Romain et al., 2020) and lead to high levels of drop-out (Firth et al., 2015). A more thorough understanding of how interventions can be supported in the real world may be helpful. In particular, whilst we know that social support is fundamental to initiation and maintenance of physical activity in people with psychosis (Carney et al., 2017; Firth et al., 2016; Soundy et al., 2014; Ussher et al., 2007), we need to better understand how caregivers support the efforts of patients to be physically active. This study aims to develop a better understanding from caregivers about what enables and prevents physical activity amongst their friends and family who have psychosis.

Typically, caregivers of people with schizophrenia in the UK spend many hours caring for their loved ones, with 43% spending over 32 hr per week (Andrew et al., 2012). They are uniquely placed in the care and support structure, in that they are likely to be a more consistent presence, spanning moves between clinical teams, services and systems and as such provide a continuity that is often missing in the NHS. They are likely to have access to a deeper knowledge about the person with psychosis; what drives them, their pre-morbid interests and abilities, and what other informal support structures could be in place to support that person in the future. When these caregivers are asked about what treatment outcomes they value most for their loved ones, physical activity is ranked highly (Lloyd et al., 2017).

Given this investment in increasing physical activity, coupled with the potential to be highly influential in patients’ care, caregivers represent a valuable resource to support physical activity interventions. However, very little research has been conducted to establish the views of caregivers when it comes to increasing the amount of physical activity undertaken by people with psychosis.

To our knowledge, there are no studies specifically addressing carers’ experiences of supporting physical activity (neither exercise nor sedentary behaviour) specifically in people with psychosis. Carer’s perspectives were addressed by Martland et al. (2021) in a qualitative investigation of high intensity interval training (HIIT) on wards, reporting predominantly but not exclusively about patients with psychosis. Carers were generally positive about the introduction of HIIT but had some concerns about the way in which it might be delivered. However, the study did not address how the performance of HIIT on wards might affect carers themselves, or what role they might play to support physical activity on wards or after discharge. Carney and colleagues (Carney et al., 2017) interviewed patients, parents and clinicians of people at ultra-high risk of psychosis about barriers and facilitators to a healthy lifestyle, including physical activity. Data from each group of participants were synthesised providing overall themes and recommendations, but not highlighting issues particular to caregivers.

Using qualitative data from 51 patients, carers and staff, a framework for increasing exercise and decreasing sedentary behaviour in people with psychosis was developed (Diamond et al., 2024). Five factors were found to promote movement and physical activity. Patients must be able to find a purpose to moving which is meaningful to them (Factor 1: Purpose). Patients need to have an expectation of the positive consequences of movement and physical activity, which can be influenced by others’ expectations (Factor 2: Predictions). A patient’s current physical (e.g., pain) and emotional state (e.g., distress about voices) needs to be addressed to allow movement and physical activity (Factor 3: Present state). Movement and physical activity can also be encouraged by the availability of effective and tailored support, provided by engaged and supported people (Factor 4: Provision). Finally, through the identification and interruption of vicious cycles (e.g., between inactivity and mood states) more positive cycles can be put in place (Factor 5: Process). Exploring carers’ perspectives is crucial to the understanding of physical activity and how to support it, and contributed significantly to the framework (Diamond et al., 2024), but a deeper analysis of carer-specific issues is warranted.

The aim of this study was to understand carer views on increasing physical activity, both in terms of increasing exercise and decreasing sedentary behaviour. These views need to be taken into account when designing and implementing interventions to help people to be more physically active.

## Material and methods

### Study design

The study was designed from a critical realist perspective. This realist/subjectivist position postulates that there is an objective reality, but that this reality is observed through the researcher’s unique lens. The data were analysed using reflexive thematic analysis (RTA) (Braun & Clarke, 2021). RTA is a qualitative research method used to identify and interpret patterns of meaning across a dataset. This method was a good fit for combining peer and non-peer researcher perspectives, and for analysing data from focus groups and interviews together.

The study was designed and conducted in collaboration with people with lived experience and experience of caring, including an Expert Advisory Group and a peer researcher. Including this perspective facilitates greater depth and nuance in data collection and analysis, and increases the trustworthiness of the findings (Gillard et al., 2010; Harding et al., 2010). Criteria for demonstrating credibility in qualitative research (Yardley, 2008) informed the study throughout (see Supplemental Appendix).

Carer participants took part alongside patients diagnosed with non-affective psychosis and staff. This paper focusses specifically on the data provided by carers: the perspective of others are reported elsewhere (Diamond et al., 2024).

The study was reviewed and approved by the NHS Health Research Authority Wales Research Ethics Committee (REC reference: 21/WA/0285) on 30.09.2021. All participants gave their informed consent prior to their inclusion in the study.

### Participants

Participants were recruited via three mental health trusts in England (Oxford Health NHS Foundation Trust, Berkshire Healthcare NHS Foundation Trust, and Humber Teaching NHS Foundation Trust). They were identified by mental health trust staff or carer support groups associated with the trust. All participants met with the researcher online or over the telephone prior to taking part and this meeting included an introduction to the researcher and the study.

Inclusion criteria for care-giver participants were: aged 16 years or older; family, partners or close friends of a person who has a clinical diagnosis of schizophrenia spectrum psychosis and experience of psychosis in the past 2 years; has at least 5 hr of caring contact per week.

Purposive sampling was used to maximise variation in participant characteristics, including age, gender, ethnicity, and relationship to the patient they cared for. The characteristics for sampling were informed by the Expert Advisory Group.

### Procedure

Focus groups (five online, one in person) were conducted by a clinical psychologist (RD). Four of these were co-facilitated by a peer researcher (AH). Focus groups included a mixture of patients, carers, and staff, allowing interactions between people in the group to contribute to learning. Five interviews (three in person, two online) were conducted by RD.

The semi-structured topic guide (see Supplemental Appendix 1), developed in consultation with the PPI Expert Advisory Group, covered sitting, standing up to break sitting time, exercising, and how people can be encouraged to move more. It was employed flexibly, to allow for the development of new ideas during participation. Follow up questions and prompts were used as needed.

Field notes and audio-recordings were taken, transcribed verbatim, deidentified and pseudonymised.

### Analysis

Analysis was guided by the six steps provided by Braun and Clarke (2021). Following familiarisation, initial, predominantly semantic, codes were created incorporating units of meaning in the whole dataset. Following analysis of the total dataset (i.e., patients, carers, and staff) together, the codes pertaining to carer issues were separated. Every piece of data within these codes was re-read as a part of the code, and then each transcript including carer participants was re-read in detail, focusing especially on the issues of caregiving. This process resulted in the addition of some new codes. The list of all codes was then discussed with members of the research team. New thematic clusters, based just on carer issues, were generated, discussed and re-generated iteratively, through a process of reflection and immersion in the data. This resulted in four themes. Discussion within the research team, including consultation with a carer advisor, resulted in the generation of a fourth theme, recognising the impact of patients’ physical inactivity upon carers.

Analysis was supported by NVivo 12 (QSR International Pty Ltd, 2018).

## Results

Between 17th December 2021 and 1st June 2022, 14 care-givers took part. Seven took part via online focus groups, two via in-person focus groups, two via interview online and three via interview in-person. Throughout the data collection, there were many important issues raised relating to caring for people with psychosis. For the purposes of this paper, we focus only on those relating to physical activity and sedentary behaviour.

Participant characteristics are presented in Table 1.

**Table 1.** participant characteristics.

Characteristic	*n*	%
Age range (years)		
16–25	0	(0)
26–35	1	(7)
36–45	0	(0)
46–55	2	(14)
56–65	7	(50)
66+	4	(29)
Gender		
Male	3	(21)
Female	11	(79)
Ethnicity		
White British	13	(93)
White other	0	(0)
Black African	0	(0)
Pakistani	0	(0)
Bangladeshi	0	(0)
Other Asian	1	(7)
Other mixed background	0	(0)
Rural or urban		
Rural postcode	3	(21)
Urban postcode	11	(79)
Carer relationship to patient		
Parent/step parent	12	(86)
Spouse/partner	1	(7)
Child	1	(7)
Team type		
Early intervention service	5	(36)
Adult mental health team	9	(64)

Four over-arching themes describe the desire to help increase physical activity in patients who were friends and family. The first describes how much the physical activity of friends and family matters, and the other three representing a call to action from carers to mental health professionals and services.

### Theme 1: Physical inactivity matters

The impact of a patient’s physical inactivity on their friends and family was multi-faceted. The potential consequences of inactivity on patients’ health was often a real concern.

I don’t want to be too dramatic, but it does seem like a slow suicide personally. It seems like “I don’t look after myself, not only do I not do exercise, but I don’t move, I eat too much,. . . it’s kind of. . .. I label myself as not being able to do stuff. It’s kind of like I set up a whole system, where I almost rejected the positive things of society” – Nick [about patient]

It makes it very difficult as a carer to just be sort of supervising decline, which is what I feel - Hugh

In some cases, the attempts to help someone to be more active were frustrating and led to less activity on behalf of the carer.

For a long time, she wouldn’t even go for a walk with me, so I’d go walking the dog, I’d sit and wait for like half an hour, so I’m going in 20 minutes, and after 20 minutes she still wasn’t out of bed. . .and it was a patience thing, and on many occasions I just gave in and said ‘all right I’m going for a walk anyway, I can’t sit here any longer’ - Sarah

We’ve been invited by a few groups of friends to go and stay somewhere where we can go for a walk. Well, [place] for example; if we go there, then she wouldn’t move off out of the accommodation, so that means I don’t do it either, which is unfortunate - Hugh

A person’s illness, and their inactivity as part of this, can result in social isolation for both the patient and their caregivers. This isolation may further constrain opportunities to be physically active.

One of the features of somebody being mentally ill is that all the people you think you can rely on just disappear out of your life. So, we’re very isolated even we’re around in a village where we know lots of people. . ... So, if we could. . . If it was possible to rebuild that network and go and see different people and different people take her for a walk – Hugh

Attempts to help on behalf of the patient can be frustrated and the process of pursuing support can feel like a battle, leading to exhaustion for carers.

What I’ve seen is staff who are so ground down that they just go for the easiest option and then because I’m on my own, I’ve got no-one to back me up when I offer any alternatives. . .. So, my own situation is I feel that certain habits have now got engrained that needn’t have got engrained if early on people had been more willing. You know I remember writing to one of the hospitals and saying, “I can fundraise with you to set up a gym room,” and I got no response. Just over a period of time, you kind of give up because you feel that when you’ve been enthusiastic and put time and energy in, the kind of responses. . . and that’s why I’m saying I can only think it’s because staff are so exhausted themselves. Because now I’m exhausted, I know how it feels – Celia

Feelings of powerlessness and exhaustion were described, resulting in carers being less available to energetically encourage activity.

I think an awful lot of mothers, parents, carers, they get a bit tired of it all and you run out of energy – Anne

But if he hasn’t got to get up, he’ll just wallow in his room and I let him wallow there because I need a break. But then he’s not benefitting – Monica

### Theme 2: Tell us

Although carers conveyed their motivation to help increase physical activity, they often reported that they do not know enough to help their friend or family member to move more. Some caregivers highlighted that although it is widely known that exercise is beneficial, the effects of sitting for long periods of time are relatively unknown.

I think it would be really interesting to do just like an online learning thing about the benefits of not sitting, or the not benefit of sitting. I think it would be really interesting. Even as a family, you know, like I said my husband has got no idea, people just don’t know, do they? Because those messages haven’t reached them. . . - Bev

Participants also described difficulties in knowing what the right thing was at any one time.

I know kind of when to push my dad and when not to push my dad. My step mum doesn’t because she doesn’t have my background. I suppose if I didn’t have that background yes, you wouldn’t know whether to push or whether not to push. And I think that’s where my step-mum is at, she like oh I don’t know. Also she doesn’t kind of know when he’s really unwell or not so again I think that’s that kind of education, not education, of knowing that this kind of is when my dad’s not doing so great. Yes, so I think a bit of education of knowing, and how to approach kind of encouraging people to exercise and things like that – Gina.

Carers also described lacking knowledge about techniques to help:

I know that, to help Vanessa, I need to know more. I need the right techniques, I need the right. . . I can’t even attempt to do your job for you, although I do think I might qualify for a Masters degree in psychology at some point [laugh] - Hugh

The provision of good quality information at important points in care was highlighted as a solution.

When you get. . .go into the Trust and you go into the system for want of a better term, you know, one of those discussions should be had that, you know, try and keep on top of. . ..getting into that. . .that. . .that sort of rabbit hole because it’s not a good place to be. And . . ..and that’s, you know, I wish that’s something we’d been told. Um, cos we were actually told give her a safe room well that’s great but she’s spent . . .but now she spends too much time in there and we’re struggling to get her out of it now. Um, so you know, a bit more balanced advice would have been. . .would have been more useful - John

Furthermore, information that is provided, should be a format that is accessible to carers. Participants illustrated that the language used is key:

The Carers Support Group that I sit on I’ve probably got more knowledge of information from there than any of the ‘ologists, no disrespect, that I’ve spoken to, um, in the Trust because it’s lived experience . . ..from people in similar positions and they can put it in smaller words enough for me to understand rather than big technical medical words - John

Participants also highlighted the importance of knowing what to expect in terms of progress for the patient. Change in recovery and in physical activity levels might not necessarily be steady and might have sudden jumps in progress:

It’s not a marathon by the end, to the week. . .it’s definitely, slow, steady, and up-and-down, very much up-and-down. . . I think something just trips in your head and you go ‘okay I can do it’ – Sarah

If this process is not understood by caregivers, this can “*demotivate the motivator*” – Henrietta.

### Theme 3: Listen to us

Caregivers have longstanding and important relationships with patients, and have usually known the person in different stages of health or across the course of their psychosis. This deep knowledge of someone gives carers a unique perspective on their current experiences and the things that may succeed or fail in helping people to be more physically active. The call to action is therefore to listen to the caregiver’s voice.

Carers frequently expressed frustration that services couldn’t see the person as they could themselves. This may include frustration that people couldn’t recognize the problems someone was facing or couldn’t see beyond the illness to the person themselves.

Talk to him like a normal person, that’s the most important thing. . . Yes, don’t patronise him, he’s not stupid and smile at him. . .Don’t treat him like an invalid [laughs] – Valerie

My son was in [hospital], and of course, they didn’t know. They only know what the doctors, nurses. . .they only see him when he was ill. Exactly what you are saying, they don’t know the person, they only see them when they are not right. So it’s important for us to fill in the gap. Because we as a mother have seen them, right from a baby. It is very important to have that background knowledge of what this person. . .they are dealing with, that’s very important. . .I don’t know when it happened. . .oh yes, I think there was one doctor who asked me ‘what was he like?’ I thought that was very good. – Jasmin

Without this knowledge it is very difficult for services to support people in the optimal way toward being more physically active.

But I don’t know how much she knows and something that came back was that she didn’t know that he had been very sporty. And that’s the sort of information which is really good for care coordinators to have – Anne

From my experience, I know my daughter inside and out, and even I found it hard, so dealing with strangers, dealing with people you don’t know, from the nurse’s point of view, must be incredibly hard. You don’t know what your starting point is, you don’t know where you’re trying to get back to - Sarah

The sense of not being listened to gave caregivers feelings of powerlessness and desperation, and negatively affected their mental health.

I experienced the same thing, that no one listened to me. . . that was the hardest time in my life. And in the end, he was. . .he became an inpatient after being sectioned. Now, that, is a horrible, horrible thing, being sectioned. . .that shouldn’t have happened. - Jasmin

### Theme 4: Ask us

Participants described a sense of wanting to be involved in the care of their loved ones, including helping them to be more physically active. Despite this, there was a feeling that their help or expertise was under-used. The call to action is therefore that professionals and services should ask for the involvement and help of carers more often.

Some carers described a sense of being distanced from the patient’s care and not knowing what work was being done. Whilst patient confidentiality can be a good reason for this, and is well-understood, it can result in carers being unaware of gaps in a service’s knowledge of the patient. Patient confidentiality is accepted as a reason for staff not to inform family members or loved ones about the care of the patient. However, this can also hinder communication between the carer and staff member in the other direction that is, staff often don’t ask for information where it could be very helpful.

In addition to the provision of information, carers also felt that their help and support could be used in other ways, but having this ignored or rejected was demotivating.

I remember writing to one of the hospitals and saying, “I can fundraise with you to set up a gym room,” and I got no response. Just over a period of time, you kind of give up because you feel that when you’ve been enthusiastic and put time and energy in, the kind of responses. . . and that’s why I’m saying I can only think it’s because staff are so exhausted themselves. Because now I’m exhausted, I know how it feels – Celia

## Discussion and conclusions

Our study confirmed that physical activity for people with psychosis really matters to carers. Yet carers’ perspectives on increasing physical activity for people with psychosis is rarely heard by services. We found a common thread of how carers felt they could potentially contribute to increasing the activity of their loved ones. Greater recognition is needed for the contribution of carers to inform future intervention efforts. For instance, services could actively support carers to be better-informed of things they could do to help, and consult with them to develop a much more personalised strategy to help an individual increase their activity. Services could also tap into carers’ motivation and knowledge, combining this with healthcare staff expertise to enhance physical activity levels.

The opportunity for carers to contribute to personalized care was a recurring theme in our findings. Research focused on increasing physical activity in people with psychosis has often highlighted the need for interventions to be tailored to an individual’s preferences and capabilities (Firth et al., 2015; Firth et al., 2016; Ussher et al., 2007; Vancampfort et al., 2012). The 5 P framework (Diamond et al., 2024) identifies that understanding a person’s purpose for moving is crucial in motivating them to get up and do an activity, but discovering the most motivating purpose can be challenging; personalising support can be challenging to perform when the delivery of the intervention is based upon limited knowledge of who a person really is, their preferences, skills and motivators. With their typically long-standing and close relationship, carers can be uniquely placed to contribute to this personalized understanding, particularly at times when articulating this is difficult for patients. This requires staff to feel empowered to seek and receive information from carers that will support direct patient care, and recognize that respecting patient confidentiality should not preclude this information sharing. Indeed, guidance from the Royal College of Psychiatrists states that ‘information sharing for the purpose of direct patient care—assisting assessment, treatment and the maintenance of safety–is encouraged’ (Royal College of Psychiatrists, 2005).

The unique knowledge a family member or close friend has about a patient not only allows them to inform interventions, but also gives them the opportunity to observe the effect of interventions by identifying even small changes in that individual’s behaviour. Moreover, by providing the patient with positive feedback to a small change, the carer can reinforce the desired behaviour change. This pattern of carers recognizing positive change (albeit discontinuous) and reinforcing it through the celebration of small but incremental improvements, is described in relation to recovery more generally (Shiers et al., 2009). However, this set of responses may not be intuitive to all carers and may need to be learned in order to have the greatest effect. Similarly, carers may themselves be subject to changes in motivation to support physical activity of their family or friends. Services can help maintain the carer’s motivation by recognizing their unique contribution, and explaining the likely discontinuous nature of improvement, thereby encouraging them to continue their support, rather than giving up.

Where appropriate, a closer working relationship between services and carers could support better intervention outcomes in relation to increasing physical activity in people with psychosis. Carers’ knowledge about the benefits of physical activity and sedentary behaviour may not be strong, nor might they be aware of what support is available locally to encourage physical activity. Empowering carers with this knowledge allows them to direct their own practical day-to-day support of their loved one, and enhance interventions offered by staff and services.

Clinical recommendations are provided in Table 2.

**Table 2.** Clinical recommendations.

Recommendation
Provide accessible information to carers which covers
The benefits of movement/impacts of sedentary behaviour for example, videos, podcasts etc., available to patients and carers.
The course of change/recovery to maintain carer motivation.
Practical techniques to support movement and when to apply them; including how carers can reinforce small positive changes incrementally.
What is available locally to promote physical activity for example, leaflets describing local community resources that encourage walking, swimming, gyms, dancing, gardening etc.
Involvement of carers in healthcare
Involve carers in care planning support for healthy levels of physical activity in patients.
Develop more personalized interventions by inviting information sharing from carers that draw on their in-depth awareness about who the patient is, their physical activity preferences or skills, and likely routes to success.
Consultation with carers about how they could enhance interventions being delivered by staff; include discussion of practical ways carers can help support between appointments.
Support for carers
Encourage carer groups to discuss physical activity.

Table 2 shows clinical recommendations to increase the impact of carer support.

There are a number of study limitations. Participants were recruited from only three sites across the UK. Recruitment was only via mental health trusts, and therefore participants were likely to be caring for people who have more severe psychiatric difficulties than those managed entirely within primary care. Although the sampling strategy was intended to maximize variation, the majority of participants were white, female, over 55 and parents of someone with psychosis, with few participants occupying other caring relationships. The study didn’t include formal carers, for example support staff at supported living accommodation. This perspective is important to integrate into future research about supporting movement. Participants all volunteered to participate and as such, are likely to have had an interest in physical activity and a very active caring role. It is important to note that for these reasons they will not be representative of all carers.

This provides one of the first insights into carers’ perspectives on physical activity for people with psychosis. It is clear that carers occupy a unique role in facilitating physical activity. The analysis presented here demonstrates that they can be neglected as key informants for services, particularly for the development and delivery of personalized care. More fulsome involvement of carers could lead to better physical activity outcomes for patients.

## Supplemental Material

sj-docx-1-isp-10.1177_00207640241277166 – Supplemental material for Supporting movement and physical activity in people with psychosis: A qualitative exploration of the carer perspective Supplemental material, sj-docx-1-isp-10.1177_00207640241277166 for Supporting movement and physical activity in people with psychosis: A qualitative exploration of the carer perspective by Rowan Diamond, Felicity Waite, Anne-Marie Boylan, Alice Hicks, Thomas Kabir, David Shiers and Daniel Freeman in International Journal of Social Psychiatry
