# Supplementary material for: Supporting movement and physical activity in people with psychosis: A qualitative exploration of the carer perspective
Source: Int J Soc Psychiatry. 2024 Sep 4;70(8):1525–32. doi: 10.1177/00207640241277166 (PMC11528945; doi:10.1177/00207640241277166)
Supplement: sj-docx-1-isp-10.1177_00207640241277166 – Supplemental material for Supporting movement and physical activity in people with psychosis: A qualitative exploration of the carer perspective [file sj-docx-1-isp-10.1177_00207640241277166.docx]

**Supporting movement and physical activity in people with psychosis: a qualitative exploration of the carer perspective**

**Supplementary materials**

**Contents**

[**Topic guide** 2](#_Toc152853035)

[**Situating statement** 2](#_Toc152853036)

# **Topic guide**

- Why do people sit down for long periods of time without getting up?
- How does the way we are feeling affect how much we sit?
- What thoughts keep you sitting down?
- What thoughts put you off standing up?
- Are there any other things that might run through your head that might make it harder to get up from sitting or lying down?
- How could any of us here do something about these problems?
- What sorts of things might help us to stand up more often?
- How does the way we are feeling positively affect our choice to stand up more?
- Are there any other things that might run through your head that might make it easier to get up from sitting or lying down?
- What helps or hinders giving support to people to be stand up more?
- What sorts of things stop people from exercising?
- How does the way we are feeling affect our choice to exercise?
- What thoughts that run through your/someone’s head might prevent you/them exercising?
- Are there any other things that might run through your head that might make it harder to exercise?
- How could any of us here do something about these problems?
- How could any of us here do more to encourage people to exercise?
- What sorts of things might help you/patients to exercise more often?
- How does the way you/patients are feeling affect the choice to exercise?
- Are there any other things that might run through your head that might make it easier to start exercising?
- What helps or hinders giving support to people to exercise?

# **Situating statement**

As clinical psychologists, RD, DF and FW viewed the data from a theoretical perspective implicit in their clinical and research work; the cognitive paradigm. A particular focus on psychological processes shaped the data collection and analysis. Data were also viewed (and collected) through the lens of lived experience (patient and carer) by the members of the Expert Advisory Group and the peer researcher.
